# The differential impact of severe childhood trauma on emotion recognition in males and females with first-episode psychosis

**DOI:** 10.1192/j.eurpsy.2021.443

**Published:** 2021-08-13

**Authors:** D. Penney, M. Pruessner, A. Malla, R. Joober, M. Lepage

**Affiliations:** 1 Psychology / Psychiatry, Université du Québec à Montréal and the Douglas Mental Health University Institute, Montreal, Canada; 2 Clinical Psychology, University of Konstanz, Konstanz, Germany; 3 Psychiatry, Douglas Mental Health University Institute, Psychiatry, Canada

**Keywords:** psychosis, social cognition, sex differences, childhood trauma

## Abstract

**Introduction:**

Childhood trauma increases social functioning deficits, which in turn negatively impact social inclusion in those experiencing first-episode psychosis (FEP). Associations between aberrant higher-order social cognitive processes such as emotion recognition (ER) and trauma severity may be one pathway by which trauma negatively impacts social functioning.

**Objectives:**

Given sex differences identified in the experience of childhood trauma, it is pertinent to evaluate how trauma severity may differentially impact ER in males and females.

**Methods:**

Eighty-three FEP participants (52 males, 31 females) and 69 nonclinical controls (49 males, 20 females) completed the CogState Research Battery. FEP participants completed the Childhood Trauma Questionnaire. A sex × group (FEP, controls) ANOVA examined ER differences and was followed by two-way ANCOVAs investigating the effects of sex and childhood trauma severity (none, low, moderate, severe) on ER and global cognition in FEP.

**Results:**

FEP participants had significantly lower ER scores than controls (p = .035). In FEP, a significant interaction emerged between sex and childhood trauma severity (F(3, 72) = 6.382, p = .001), selective to ER, while controlling for age at onset. Simple effects analyses revealed that females in the severe trauma category exhibited superior ER capacity relative to males.

**Conclusions:**

The differential impact of trauma severity on ER in males and females with FEP may be theoretically interpreted as the distinct way that hypervigilance affects the sexes. Early intervention services should refine social cognitive interventions in male and female trauma survivors to facilitate social functioning improvements.

**Disclosure:**

Funding: This study was supported by the Canadian Institutes of Health Research (#68961) to M.L. and A.M. Salary awards include: The Fonds de recherche du Québec - Santé to M.L. and R.J., the James McGill Professorship to M.L., and the Canada Research Cha

